# Shared decision making in the social services? Reasons to consider when choosing methods for service user participation

**DOI:** 10.1111/jep.13323

**Published:** 2019-12-02

**Authors:** Pia Nykänen

**Affiliations:** ^1^ Department of Education, Communication and Learning University of Gothenburg Gothenburg Sweden

**Keywords:** reasons, shared decision making, social care, social services, social work, user participation

## Abstract

User participation is nowadays a desirable feature of social services work. The International Federation of Social Workers states that staff shall promote the participation of clients so as to “enable them to be empowered in all aspects of decisions and actions affecting their lives.” The statement is codified in various national ethical codes; the Swedish Code of Conduct and Ethical Behaviour for Social Workers specifies that interventions shall build on client participation and common agreement. However, a 2012 Swedish governmental report noted that among 16 methods for user participation in the social services, psychiatry, and abuse and addiction care, only one, shared decision making (SDM), had been evaluated in randomized controlled trials (RCTs). Given this lack of evaluations, how ought professionals to choose between the various methods? The aim of this article is to introduce distinctions in order to answer the question of how social workers ought to choose between different user participation methods, to suggest how this choice could be made, and to argue that the case for SDM seems to be stronger than for other methods. We can distinguish between justificatory, motivational, and explanatory reasons in order to clarify what types of reasons are relevant when choosing between methods. Another distinction concerns general and specific reasons for user participation. No particular method for user participation can inherit its support only from general reasons, since these ordinarily do not point out any method as better than another one. Rather, specific reasons are needed. Social workers do have good reasons for choosing certain methods for user participation rather than others. These methods can be found by looking at specific justificatory reasons. The case for SDM is strengthened by its having been evaluated in RCTs and also because the SDM components harmonize with relevant components in the presented (Swedish) legislation.

## 1 INTRODUCTION

In several countries, service user participation, or service user involvement, is considered a desirable feature of social services work. International and national guidelines and recommendations instruct the social services to perform their work with clients so that service user participation is realized. In their “Statement of Ethical Principles,” the International Federation of Social Workers (IFSW) states that “[s]ocial workers should promote the full involvement and participation of people using their services in ways that enable them to be empowered in all aspects of decisions and actions affecting their lives.”^1^ The international statement is codified in various national ethical codes for the social work profession. For instance, the Swedish Code of Conduct and Ethical Behaviour for Social Workers tell the Swedish professionals that interventions, as far as possible, should “build on client participation and common agreement.”^2^

At the same time, in Sweden as well as in many other countries, there is an increasing demand that the social services work in accordance with an evidence‐based practice (EBP). The professional should systematically take into account the best available knowledge and/or research; his or her own expertise; the client's or service user's situation, experience, and preferences; and external circumstances (such as legislation, guidelines, and locally available interventions).^3^

Much research has been concerned with the possibilities and limitations of EBP, and the Swedish debate has largely focused on the governmental authorities' top‐down approach in directing social work practitioners to implement EBP. As a consequence, the question of desirable service user participation methods has tended to become somewhat invisible. In 2012, a governmental report noted that among 16 methods or models for service user participation in the Swedish social services, psychiatry, and abuse and addiction care, only one, shared decision making (SDM), had been evaluated in randomized controlled trials (RCTs).^4^ Among the 16 were methods aimed at the organizational level, such as different “user audits” (for instance, BuKu and The Involvement Model [IMO]) and methods concerned with the individual user level, such as Life Stories, Personal Ombudsman, and SDM. This article deals primarily with SDM, and therefore, the SDM procedure's nine practical steps that the professional and the user should engage in are described below.*, 5

Even without the benefit of thoroughly evaluated participation methods to choose from, the social services clearly aim to work in a user‐friendly way, we can find many methods and initiatives for user participation in the Swedish social services and also internationally. Yet what influences the choice of methods may, as an example, be local pull factors such as colleagues from a neighbouring municipality's social services speaking warmly about a particular method. Institutional isomorphism and mimetic processes have been thoroughly described by researchers such as DiMaggio and Powell.^6^ Organizations mimic each other for a number of reasons, such as legitimacy and trustworthiness.

Given that there are many different user participation methods but that few of them have been evaluated, how ought staff to choose between them? Do professionals in the social services have good reasons for promoting or choosing certain methods for service user participation rather than others? What questions need to be asked in order to qualitatively distinguish between different methods? Are there any yardsticks to be found that can help us in assessing the methods?

The main purpose of this article is to help us think more clearly about reasons for different methods of service user participation. The “us” in the former sentence refers to anyone interested in user participation in the social services, but hopefully, this will be of particular interest to professionals and researchers in social work. This purpose will be achieved by introducing distinctions in order to answer the question of how social workers ought to choose between different user participation methods and also by suggesting how this choice could be made.

The normative point of departure is that we should ascribe weight to evaluations of user participation methods (and the results of these evaluations) when deciding which method we should choose. Several assumptions are made here, for example, it is possible to give rational reasons for why one (or a group of) method(s) should be chosen rather than another (or others), and secondly, it is desirable to give rational reasons for these choices. Another assumption is this one: the fact that a method has been evaluated is a good thing (whether the participation method in itself is good or whether the design of the evaluation is good, is a different matter). The following assumption is made as well: quality of the design of evaluations is a matter of degrees.

A second point of departure is that even if we cannot find any evaluations of methods, this does not mean that “anything goes,” that is, that all methods are equally good (or bad). The suggestion made here is that even if evaluations are (or would be) absent, we may find other reasons that support (or do not support) our choices.

Some reservations are needed: the assumption that quality of design of evaluations is a matter of degrees, does not in itself presuppose a particular system for “evidence grading.” Neither does the article want to argue that decision making is an easy and clear‐cut matter, whether at the individual level or the organizational. Decisions in the social services are often distributed across time, people, and different levels of the organization.^7^

Finally, the article will also argue that the case for SDM seems to be stronger than for other methods.

Now, are these issues important, and if so, why? We may argue for their importance in the following way: (a) Under prestige words such as “user participation” and “user influence” hide working modes that sometimes do not have much to do with participation or influence at all, or that only entail low‐level participation (even when the circumstances do not provide good reasons for this low‐level participation). (b) We should strive for well‐founded ways of working in this area as well as in other areas. There are no good reasons for treating user participation poorly, rather the opposite, since user participation in the social services may be of utmost importance to both users and staff. (c) This arena is sensitive to trends that might influence the professional's decision unduly.^8^ We need ways to assess the methods promoted by entrepreneurs and innovators at in‐service training courses and conferences for social service professionals. Making money by selling one's “own” participation method is not a bad thing per se, but it is a problem if that method is qualitatively bad or worse than other methods. (d) We should try to increase the robustness and prevalence of user participation (where it is effective). If the choice of a particular method at a social service workplace is due perhaps primarily to one person's enthusiasm for it and not the reasons for the method itself, then its survival is vulnerable in the long run (and this is a bad thing if it is a good method).

Some introductory comments are necessary before looking at the different kinds of reasons. Firstly, someone might question the situation presented in the title of this paper and claim that professionals never find themselves weighing different reasons for and against different methods. Rather, it is a question of what method(s) is/are promoted and chosen by the social services management. Secondly, one might object to the fact that different initiatives for user participation are being presented as “methods.” There are different ways of working, but there are no standardized participation models as the word “method” suggests.

Both objections are valid. However, even if it could be shown that professionals never weigh reasons for and against different participation methods, that does not mean that they (or the social services management) should not do it. As for the words “method” and/or “model” (they are here used interchangeably), they refer in the article to all those ways of working with service user participation or service user involvement that are to be found in social work research, steering documents for the social services, local action plans, etc. References to “method” or “model” therefore include instances such as deliberative workshops, feedback‐informed treatment (FIT), journey mapping, SDM, surveys and many more.

While children's participation is an important and interesting issue concerning the social services, this question is not treated in this article; “service user” here refers to an adult client.

## 2 GENERAL REASONS FOR USER PARTICIPATION

There are many kinds of reasons. They can be categorized, for example, into reasons of justification, motivation, and explanation.^9^ Here, we are not looking for explanatory reasons but for justificatory reasons. “A good reason” here is thus to be understood as something like “a good argument.”

Sometimes, the *purpose* of service user participation is stressed, however, perhaps most often in grey literature.† The emphasis on purposes in this literature may boil down to there being lots of participation initiatives at the local level, but sometimes, these are orchestrated without a clear purpose. “Take stock: be sure why you should do it at all” is emphasized in one guiding document for local workplaces.^12^ In this article, “purpose” refers to these local aims or goals for service user participation and should not be confused with the justificatory reasons that are being explored.

An implicit premise for promoting user participation is that user participation is desirable. But why and how is it desirable?‡ A pertinent question is whether user participation is viewed as instrumental (as a means to an end) or intrinsic (as an end in itself). Among the several general justificatory reasons for the social services to work with user participation, many ascribe value to user participation in virtue of its being a means to something else that is considered good. These reasons are often of a moral kind, such as arguments that have to do with promoting user autonomy and empowerment. But there are also arguments related to the knowledge or expertise of service users. User participation is then considered desirable because of the knowledge (of the user's situation, history, preferences, etc) that the client brings to the situation.

Another group of reasons are of a political kind, for instance, that user participation helps us foster democratic citizens or may help our welfare services achieve legitimacy in the eyes of the citizens.^10^ There are also economic reasons, such as cost‐reducing arguments, where user participation is viewed as contributing to the cost‐efficiency of our welfare services. Other efficiency reasons have to do with the supposition that user participation makes users more prone to adhere to or comply with, for instance, prescribed treatment (and as a consequence, perhaps recover faster). We can also find quality reasons, where user participation is a means to raising the quality of the social services.^13^

Many reasons can be combined in different ways. For instance, the quality reasons can be combined with reasons that have to do with user satisfaction. This is the case when user participation is viewed as a means to user satisfaction and where the quality of a social work agency's activities is measured in terms of service user satisfaction.

All of the above arguments need to be assessed according to validity and relevance, and many reasons are in need of empirical support. If we claim that X (a particular model for user participation) increases the sense of self‐determination, we need to show that it does or at least is likely to do so. Or if we claim that X strengthens democracy by virtue of fostering democratic citizens or raises the quality of the social services, we need to justify these claims as well; in these two latter cases, this needs to be done in relation to a particular view on democracy and a particular view on, or interpretation of, “quality.”

There are legislative and guiding arguments for user participation as well. To this group belong reasons found in a particular country's legislation and/or governmental guidelines or recommendations. The Swedish 1974 Instrument of Government (one of the documents that make up the Swedish constitution) states that the public institutions should “promote the ideals of democracy as guidelines in all sectors of society” and also “promote the opportunity for all to attain participation and equality in society.”^11^ And in the Swedish Social Services Act, we find several values that, taken together, express a particular perception of what it is to lead a good life; this perception includes components such as meaningfulness and active participation in community life. It is therefore not surprising that participation in general is promoted in the Swedish welfare services. However, there are also professional reasons, such as reasons deriving from a particular profession's code of ethics, as in the IFSW statement presented above.

Yet another group of reasons emanates from the idea of customer rights. For those who claim that service users are equivalent to customers, user participation may be viewed as a way of exercising one's rights as a customer.

However, the reasons above can be used to argue for many different user participation models. This is what makes the reasons or arguments general. If we want to argue that we should choose participation method X rather than participation method Y, we need to find good and more specific reasons that support method X.

The question of which method to choose and why actually concerns three different questions: (a) What *kind* of user participation is desirable? (b) What *degree* of user participation is desirable? (c) What *extent* of user participation is desirable? A certain method might be desirable in a particular situation for a particular client by virtue of its being of a certain kind, that it contains a certain degree of participation and that it comprises a certain area. This article deals primarily with the first question.

## 3 SPECIFIC REASONS

### 3.1 Reasons for particular methods of participation

General reasons or arguments for user participation was presented above. However, these cannot give us reasons enough for choosing one method for participation rather than another. But what about more specific reasons or arguments? Let us first consider some to which you as a social worker should not ascribe decisive weight. This group includes arguments from authority (method X is promoted by Y, where Y may be your boss, your favourite user participation theoretician or perhaps the enthusiast in your workplace), appeal to novelty (X is a new method), and appeal to tradition (X has always been our method for user participation). The list could be made much longer, but these few will suffice to make the point. That method X is promoted by Y or that X is a new method does not make X a good method and/or the method to choose.

But what about the argument that the social worker prefers method X? This will not do either, as will be shown below.

Let us first try another way to find specific reasons and test the idea of finding specific reasons by extracting these from some of the general ones. Let us turn to the legislative arguments and arguments from a code of ethics. These are relevant since they are steering and guiding documents for the social workers. We will take a closer look at the content of the Swedish Social Services Act and the Swedish Code of Conduct and Ethical Behaviour for Social Workers.

The Social Services Act establishes that the social services shall promote people's active participation in society and that the services must be based on respect for people's self‐determination and integrity. There are also formulations about quality in the Act: “Efforts within the social services must be of good quality […] there must be staff with appropriate training and experience. The quality […] must be systematically and continuously developed and secured.” And last but not least, the efforts for the individual “shall be designed and implemented together with him or her.”^14^

When we turn to the code of conduct and ethical behaviour for social workers, a number of values are enumerated. Among these are respect and integrity, liberty and self‐determination, and democracy and participation.^2^

When close reading the steering and recommending documents for social workers, we find writings having to do with the individual's self‐determination, the importance of his or her involvement, and the quality of the services. Let us now view *user participation* through the lenses of these imperatives and ask ourselves the following question: How should “good quality” be determined in relation to user participation methods? The following are some of the characteristics or properties that are conducive to good quality: staff with appropriate education and experience, legal certainty, the involvement of individual users, well‐thought‐out working methods (and here, it is specified that this also means that follow‐ups and evaluations of interventions are necessary), staff responsiveness and empathy, respect for users' personal integrity, and that users have both insight into and influence over the services they receive.^15^

We will now return to the question of whether the preferences of the professional constitute a good reason for choosing method X. It is easy to see why this reason alone will not do. The fact that one or many social workers prefer method X is not enough to ensure good quality.

The question now posed is: Do any methods for participation better live up to the values stated in the legislative and guiding documents? Naturally, the answer will be the result of an interpretative act since degrees of “fit” between method and values are open to questioning. However, the point here is that we may put certain limitations on the interpretations, that is, not all interpretations are equally good. We may suggest that determining how well method and value “fit together” entails investigating at least four things.

Firstly, what do the legislative documents actually say? Take the “good quality criteria” above. If we, as staff, are about to choose a method for user participation and we choose to establish a service user council while at the same time we do not have methods for individual user participation, this seems not to be an instance of good quality since *the individuals'* influence over the efforts or interventions concerning themselves is emphasized in the steering documents. Or if we, as staff, have not received any *education* in user participatory methods, or if we do not *evaluate* or follow up our participatory methods, this also amounts to lower quality.

Secondly, do the different values (for instance, in the Social Services Act) correlate with or support each other, and if so, how? This is a trickier matter and amounts to an almost hermeneutical act. Consider a Social Service Act containing the value “democracy”; we may differ on the meaning of that term and we also often prefer different ideals of democracy. If “democracy” means a representative democracy for A, a deliberative democracy for B, and a participatory democracy for C, these three may conceive the “fit” between value and method differently and, on the basis of this, embrace or dismiss different methods for participation. However, if the Act also states that “active participation” is desirable, we may assume that a participatory view on democracy has more support and that we should assess our participation methods in relation to this view. Now, the first and second suggestions on how to assess “fit” rely on the existence of a morally sound legislation; it is presumed here that the values inscribed are “acceptable” to us. But what about the cases when national laws and guidelines do not “cohere” at all, or do so only to a minor extent, with our moral intuitions? This issue cannot be dealt with thoroughly here, but perhaps then the “fit” between one's professional‐ethical code and the participatory methods should be assessed instead.

Thirdly, we may find preparatory works that can help us interpret our legislation. A Swedish example is found in some of the government bills preceding the Social Services Act. There, it is expressed that the individual should have “a real influence” over the services received.^16^ Such a statement supports a participation method that includes a high *degree* of participation (“real influence”), but it also says something about the *extent* (influence over the efforts or interventions). Any one participatory method cannot be more “real” than another; rather, we should here think of different degrees and extents of participation.

Lastly, the “fit” between method and value is something that the social workers themselves should assess as well, since “fit” between method and value is also a matter of professional judgement. This means that there are limitations on who is suitable for determining “fit.” Social workers make assessments of the “fit” based on their knowledge of the individual users and/or the target groups they are about to meet. Naturally, the answers to questions of what kind, what degree, and what extent of user participation is desirable are not to be found solely in texts. Professional judgement is imperative when it comes to finding these answers; however, it is not sovereign when it comes to determining the “fit.”

Evaluation of methods or interventions is part of what constitutes “good quality,” according to the above. This points in favour of those methods for participation that actually have been evaluated. But what sort of evaluation of participatory methods is desirable? If the individual users should have influence over the services provided, this opens up for at least two different forms of evaluation. One is to investigate whether users *feel* that they have had influence; another way is to find out whether they *actually had* influence. One might feel involved and yet not have had much influence at all over the services received. If the legislative documents emphasize “real influence” (as the Swedish government bill does), this seems to point in the direction of the second suggestion.

### 3.2 User preferences as reasons and reasons for SDM

As noted above, the preferences of the professional do not constitute a good reason for choosing method X, given the “good quality criteria” stated above. But what about the *user's* preferences? Let us turn to this argument: participation method X is preferred by the user concerned. Remember that the Swedish Social Services Act and the code of ethics do not mention participation methods per se but efforts or interventions. However, there are no good reasons for excluding real influence by the user regarding this particular area.

An interesting empirical finding related to this is the one made by Eriksson, who noted that users involved in organizational discussions on user participation had the most influence over future user participation.^13^ The organizations explained this phenomenon by noting that the forms for participation were not yet in place, but when they eventually were ready, the users would get influence over other questions.

This may be seen as curious, but let us twist this in favour of the subject at hand and return to the components in an EBP. As mentioned, the best available knowledge and/or research, the professional's expertise, the person's (the client's or service user's) situation, experience, and preferences and external circumstances (such as legislation, guidelines, and locally available interventions) should be taken into account in a systematic way. There are no good reasons for excluding the user's preferences or influence when it comes to choosing methods for user participation. Rather the opposite, since it is the client's well‐being that is and should be the main concern. The user's preferences constitute reasons to be taken into account. This means that the user should have “real influence” in this area as well. What *kind* of participation does the user prefer? *How much* does the user want to participate? And *concerning what*? One could therefore say that some sort of SDM should be applied also when it comes to choosing the desirable method for user participation.

We already know that SDM has been evaluated in RCTs, and this gives the method a certain head start compared with other participatory methods. We should primarily choose evaluated methods (given that the results are not negative). One of the “quality criteria” also emphasizes evaluation of one's methods. But then we are left with a circular argumentation; our question concerned how we ought to choose in the *absence* of evaluations. However, there seems to be a certain harmony or “fit” between the SDM components and other stated quality criteria as well; as described in the introduction, SDM is a method for an *individual*, and integrated in the nine SDM steps are components such as individual users' *involvement* and the users getting both *insight into* (via presentation of treatment options and information on benefits and risks of the options) and *influence over the services that they receive*. Investigation and identification of *the users' expectations and preferences* are also integral parts of the procedure, and finally, a *follow‐up* is included.

All methods for user participation may face dilemmas and tensions. This sort of “meta‐SDM” (when we ask about what kind of participation the user prefer; how much the user want to participate and concerning what) is no exception. Conflicts between the user's preferences and the social workers mission and preferences can—and do—occur everywhere. However, it is hoped that influence over one's participation widens the space for different kinds, degrees, and extents of participation.

Moreover, SDM is not one homogeneous method but a cluster of many versions. This is so, since the steps in the SDM process allow for different degrees. Different versions of SDM may be appropriate in different situations.^17^ Choosing SDM brings with it further new choices and assessments.

## 4 CONCLUSIONS

The aim of this article was to introduce distinctions in order to answer the question of how social workers ought to choose between different user participation methods, to suggest how this choice could be made and to argue that the case for SDM seems to be stronger than for other methods.

The distinction between justificatory, motivational, and explanatory reasons was introduced to clarify what types of reasons are relevant in the choice between methods. Another distinction was introduced as well, the one between general reasons and specific reasons for user participation. No particular method for user participation can inherit its support only from general reasons since these ordinarily do not point out any method as better than another one. Rather, specific reasons are needed.

Social workers do have good reasons for promoting and/or choosing certain methods for user participation rather than others. These methods can be found by looking at different specific justificatory reasons. The case for SDM is strengthened by its having been evaluated in RCTs and also because the SDM components harmonize with relevant components in the presented legislation.

## CONFLICT OF INTEREST

The author declares no conflict of interest.

## FUNDING INFORMATION

The project that made this research possible is funded by the Swedish Foundation for Humanities and Social Sciences.

## ENDNOTES

*
(a) Disclosure that a decision needs to be made, (b) formulation of equality of partners, (c) presentation of treatment options, (d) informing the client about the benefits and risks of the options, (e) investigation of the client's understanding and expectations, (f) identification of both parties' preferences, (g) negotiation, (h) reaching a shared decision, and (i) Arrangement of follow‐up.

†
Two examples are HSE^12^ and Eriksson^13^.

‡
The reasons enumerated in these sections are described in a schematic way. In fact, user participation and its relation to, for instance, different aspects of democracy, are far more researched than it may seem here. See, for example, works by Peter Beresford and Sarah Carr.
